# Trends in Major Risk Factors for Cardiovascular Disease Among Adults in the Mississippi Delta Region, Mississippi Behavioral Risk Factor Surveillance System, 2001–2010

**DOI:** 10.5888/pcd12.140481

**Published:** 2015-02-19

**Authors:** Vincent L. Mendy, Rodolfo Vargas

**Affiliations:** Author Affiliation: Rodolfo Vargas, Mississippi State Department of Health, Greenwood, Mississippi.

## Abstract

**Introduction:**

The prevalences of major modifiable risk factors for cardiovascular disease (CVD) are disproportionately high in the 18-county Mississippi Delta region, and many of these risk factors disproportionately affect blacks. Temporal trends in the prevalence of CVD risk factors in the Mississippi Delta have not been determined. We examined trends in CVD risk factors from 2001 to 2010 in the region.

**Methods:**

Longitudinal trends in prevalence of high blood pressure, high cholesterol, diabetes, obesity, physical inactivity, and current smoking were investigated using self-reported data from the Behavioral Risk Factor Surveillance System. Joinpoint regression models were used to examine annual percentage change (APC) in the prevalence of these risk factors.

**Results:**

Overall, from 2001 to 2010, we observed significant increases in the prevalence of high cholesterol (APC, 4.22%), obesity (APC, 3.65%), and diabetes (APC, 3.54%). Among blacks, we found significant increases in the prevalence of high cholesterol (APC, 3.41%), obesity (APC, 3.48%), and diabetes (APC, 4.96%). Among whites, we found significant increases in high blood pressure (APC, 2.18%), high cholesterol (APC, 4.78%), obesity (APC, 4.18%), and physical inactivity (APC, 3.06%). We also observed a significant decrease in smoking among whites (APC, −1.99%).

**Conclusion:**

From 2001 to 2010, we found a significant increase in the prevalence of high cholesterol, diabetes, and obesity in the Mississippi Delta. We also observed racial differences in those prevalences.

## Introduction

The 18-county Mississippi Delta region covers about 11,000 square miles of the northwest part of the state between the Mississippi and Yazoo rivers. In 2010, its population was 554,754; 49.7% of residents were black and 46.9% were white (1). The Mississippi Delta is known for its perennial poor health outcomes and has some of the most profound disparities in cardiovascular health in the state and the nation (2). Cardiovascular disease (CVD) is the leading cause of death in the Mississippi Delta (2). In 2012, heart disease (244.4 deaths per 100,000 population) and stroke (49.0 deaths per 100,000 population) were the first and sixth leading causes of death in the Mississippi Delta (3). At the national level, CVD disproportionately affects blacks (4) and is the largest cause of lower life expectancy among them (5).

Major modifiable CVD risk factors include high blood pressure, high cholesterol, diabetes, obesity, physical inactivity, and smoking (6). The prevalence of these factors is higher in the Mississippi Delta than in the non-Delta region of the state, and these factors disproportionately affect blacks (7,8). Assessing temporal trends in the prevalence of these risk factors provides useful information for needs assessment, as well as for developing and evaluating health promotion programs and policies for the target communities (9). Investigating trends and annual percentage change (APC) in the prevalence of CVD risk factors is crucial in the Mississippi Delta to address disparities in CVD and promote prevention strategies that will decrease CVD morbidity and mortality. To address a gap in this knowledge, we examined trends in the prevalence of CVD risk factors from 2001 to 2010 in the Mississippi Delta among the population as a whole and in the black and white populations. The Mississippi Delta Health Collaborative (MDHC) is a 5-year cooperative agreement between the Centers for Disease Control and Prevention (CDC) and the Mississippi State Department of Health designed to prevent heart disease, stroke, and related chronic diseases in the Mississippi Delta. The interventions target the “ABCS” (aspirin therapy, blood pressure control, cholesterol management, and smoking cessation) of heart disease and stroke prevention in the region.

## Methods

### Data source and study population

The Behavioral Risk Factor Surveillance System (BRFSS) is a state-based, random-digit–dialed telephone survey of the US noninstitutionalized civilian population aged 18 years or older. The BRFSS is conducted in all 50 states, the District of Columbia, and 3 US territories (Puerto Rico, Guam, and the US Virgin Islands). Data from the BRFSS have been shown to reliably and validly assess CVD risk factors (10,11). Detailed information about BRFSS is available at www.cdc.gov/brfss/.

Mississippi BRFSS data from 2001 to 2010 were combined for our analysis; data on 11,978 participants residing in the Mississippi Delta, for whom we had complete information on the variables of interest, were analyzed. The 18-county region includes Bolivar, Carroll, Coahoma, DeSoto, Holmes, Humphreys, Issaquena, Leflore, Panola, Quitman, Sharkey, Sunflower, Tallahatchie, Tunica, Tate, Warren, Washington, and Yazoo counties. The populations of Bolivar, Coahoma, Holmes, Humphreys, Issaquena, Leflore, Quitman, Sharkey, Sunflower, Tallahatchie, Tunica, Warren, Washington, and Yazoo are mostly black (50%–83%), whereas the populations of Carroll, DeSoto, and Tate counties are mostly white (65%–72%); Panola county has equal numbers of whites and blacks (1). BRFSS 2001–2006 sample sizes ranged from 515 to 972 respondents; to generate more reliable estimates on CVD risk factors, the region was oversampled in 2007 (n = 2,117) and 2009 (n = 2,491).

BRFSS consists of self-reported data on high blood pressure, high cholesterol, diabetes, obesity, current smoking, and physical inactivity. For high blood pressure and high cholesterol, our analyses were constrained to 2001, 2003, 2005, 2007, and 2009 survey years because questions about these measures were asked only in these years; all other variables were assessed in consecutive survey years from 2001 to 2010. Also, only participants who self-identified as black (n = 5,341) or white (n = 6,637) were included in this analysis; these racial groups accounted for 96.6% of the Mississippi Delta population in 2010 (1).

### Covariates

Covariates assessed were age, race, sex, household income, education level, and health insurance status and were categorized as follows: age in years (18–34, 35–49, 50–64, and ≥65), household income (<$20,000, $20,000–$34,999, $35,000–49,999, and ≥$50,000), education level (<high school, high school, and >high school), and health insurance status (yes and no).

### CVD risk factors

High blood pressure was defined as a yes response to the question “Have you ever been told by a doctor, nurse or other health professional that you have high blood pressure?” High cholesterol was defined as a yes response to the question “Have you ever been told by a doctor, nurse or other health professional that your blood cholesterol is high?” Diabetes was defined as a yes response to the question “Have you ever been told by a doctor that you have diabetes?” Current smoking was defined as having smoked at least 100 cigarettes during the respondent’s lifetime and currently smoking at the time of the survey. Obesity was defined as having a body mass index (BMI) of 30.0 kg/m^2^ or more (calculated from self-reported height and weight) and categorized as obese or not obese. Physical inactivity was defined as a no response to the question “During the past month, other than your regular job, did you participate in any physical activities or exercise, such as running, calisthenics, golf, gardening, or walking for exercise?”

### Statistical analyses

Data were analyzed using SAS 9.3 (SAS Institute Inc) to adjust for the disproportionate stratified sampling design of the BRFSS and were weighted using post-stratification methods (12). Logistic regression analysis was used to test for change over time in the prevalence of high blood pressure, high cholesterol, diabetes, obesity, physical inactivity, and current smoking. The regression models controlled for changes in distributions by age, race, sex, household income, education, and health insurance. The models also assessed the linear and quadratic time effect by including time variables (linear and quadratic) that were treated as continuous and were created by coding each year with orthogonal coefficients calculated using PROC IML in SAS. Adjusted prevalence and associated standard errors were calculated by year using SUDAAN 11 (RTI International) and then exported to Joinpoint software (4.1.1) from the US Surveillance, Epidemiology, and End Results (SEER) program (http://surveillance.cancer.gov/joinpoint/) to 1) determine the critical year, or joinpoint (where the prevalence trend might change direction because of a significant quadratic trend) and 2) calculate the APC for a linear or quadratic trend. Analysis were constrained to minimum joinpoints (ie, 0 joinpoint, representing a straight line) where quadratic trends were not significant. In joinpoint analysis, the best-fitting points (where the rate increases or decreases significantly) are selected. Analysis starts with a minimum number of joinpoints and tests whether 1 or more joinpoints are significant and should be added to the model (13). Trends are described by the APC (14). APCs and 95% confidence intervals (CIs) are calculated for each linear trend by fitting a regression line to the natural logarithm of the prevalence rates using calendar year as a regression variable (15). The APC is tested to examine whether a difference exists from the null hypothesis of no change (0%) (13,16). The APC is significantly different from 0 at *P* value less than .05.

## Results

Overall, the mean age of the respondents was 45.0 years; 47.0% were black, 53.3% were women, 26.3% reported a household income below $20,000, 21.5% reported less than a high school education, and 75.5% had health insurance (Table 1).

**Table 1:** Sociodemographic Characteristics of Mississippi Delta Adult Respondents, Behavioral Risk Factor Surveillance System, 2001

Characteristic	%^a^ (95% Confidence Interval) (n = 515)
**Age, y**	
18–34	33.5 (27.8–39.3)
35–49	29.7 (24.9–34.5)
50–64	21.7 (17.7–25.7)
≥65	15.1 (11.8–18.4)
**Race**	
White	53.0 (47.4–58.5)
Black	47.0 (41.5–52.6)
**Sex**	
Male	46.7 (41.1–52.3)
Female	53.3 (47.7–58.9)
**Household income, $**	
<20,000	26.3 (21.4–31.1)
20,000–34,999	22.9 (18.6–27.3)
35,000–49,999	14.8 (10.5–19.0)
≥50,000	18.0 (14.4–21.7)
No answer	18.0 (13.4–22.6)
**Education level**	
Less than high school	21.5 (17.0–26.0)
High school or equivalent	33.8 (28.4–39.3)
More than high school	44.7 (39.3–50.0)
**Health insurance**	
No	24.5 (19.1–29.9)
Yes	75.5 (70.1–80.9)

a Weighted percentage.

Overall, after controlling for all covariates, we observed significant linear increases in the prevalence of obesity (APC, 3.65%; 95% CI, 2.01%–5.32%), diabetes (APC, 3.54%; 95% CI, 0.24%–6.94%), and high cholesterol (APC, 4.22%; 95% CI, 2.24%–6.25%) (Table 2).

**Table 2:** Prevalence of, and Trends in, Selected Cardiovascular Disease Risk Factors Among Mississippi Delta Adults, Mississippi BRFSS, 2001–2010

Risk Factor	Prevalence, %^a^										Trends Determined by Joinpoint Analysis		
	2001	2002	2003	2004	2005	2006	2007	2008	2009	2010	Period	APC (95% CI)	*P* Value^b^
**Overall**													
High blood pressure	33.7	NA	35.5	NA	35.8	NA	35.2	NA	39.0	NA	2001–2009	1.55 (0.64 to 3.79)	.11
High cholesterol	30.2	NA	32.0	NA	35.7	NA	40.2	NA	41.3	NA	2001–2009	4.22 (2.24 to 6.25)	.006
Diabetes	9.2	7.8	11.5	8.7	9.6	12.9	12.1	12.1	12.0	11.2	2001–2010	3.54 (0.24 to 6.94)	.04
Obesity	30.9	28.2	29.9	29.3	34.0	36.7	38.1	38.3	37.8	37.8	2001–2010	3.65 (2.01 to 5.32)	.001
Physical inactivity	32.2	33.2	29.3	33.9	31.4	31.4	34.8	35.3	34.6	31.7	2001–2010	0.89 (−0.61 to 2.42)	.21
Current smoking	25.5	28.5	26.6	24.0	27.2	27.1	24.7	22.8	26.1	21.5	2001–2010	−1.41 (−3.28 to 0.50)	.12
**Black**													
High blood pressure	37.7	NA	40.6	NA	42.3	NA	36.7	NA	43.7	NA	2001–2009	1.01 (−3.95 to 6.23)	.64
High cholesterol	28.8	NA	27.3	NA	31.4	NA	34.6	NA	35.2	NA	2001–2009	3.41 (0.72 to 6.17)	.03
Diabetes	10.6	8.9	13.9	8.3	12.6	12.9	13.9	15.2	14.9	13.7	2001–2010	4.96 (1.03 to 9.05)	.02
Obesity	36.4	33.7	36.1	37.0	41.1	13.5	46.4	45.3	43.9	46.7	2001–2010	3.48 (1.93 to 5.05)	.001
Physical inactivity	39.3	40.4	34.1	39.4	40.0	36.6	40.5	36.3	36.4	35.1	2001–2010	−0.85 (−2.46 to 0.77)	.26
Current smoking	24.5	28.0	23.1	22.0	22.5	28.2	22.3	19.4	27.4	20.9	2001–2010	−0.52 (−4.18 to 3.29)	.76
**White**													
High blood pressure	29.7	NA	30.7	NA	29.3	NA	33.7	NA	34.7	NA	2001–2009	2.18 (0.37 to 4.03)	.03
High cholesterol	31.1	NA	35.9	NA	39.1	NA	44.8	NA	46.4	NA	2001–2009	4.78 (2.63 to 6.97)	.006
Diabetes	7.8	6.6	9.2	8.8	6.6	12.7	10.3	9.3	9.2	8.9	2001–2010	1.61 (−2.49 to 5.87)	.40
Obesity	25.5	22.7	24.4	21.9	27.1	30.0	30.2	32.0	32.2	30.1	2001–2010	4.18 (1.97 to 6.45)	.002
Physical inactivity	25.6	26.8	25.1	28.8	23.5	26.4	29.4	34.3	33.0	28.4	2001–2010	3.06 (0.50 to 5.69)	.02
Current smoking	26.8	29.4	29.3	25.8	31.7	26.3	26.9	25.9	24.5	22.4	2001–2010	−1.99 (−3.86 to −0.09)	.04

Abbreviation: APC, annual percentage change; BRFSS, Behavioral Risk Factor Surveillance System; CI, confidence interval; NA, data not collected.

a Adjusted for age, race, sex, household income, education, and health insurance status.

b APC is significantly different from 0.

A significant increase in obesity prevalence was observed among blacks (APC, 3.48%, 95% CI, 1.93%–5.05%) and whites (APC, 4.18%; 95% CI, 1.97%–6.45%) after adjusting for all covariates. Similarly, high cholesterol significantly increased among blacks (APC, 3.41%; 95% CI, 0.72%–6.17%) and whites (APC, 4.78%; 95% CI, 2.63%–6.97%). For diabetes, a significant increase was observed among blacks (APC, 4.96%; 95% CI, 1.03%–9.05%) but not whites. A significant increase in physical inactivity was observed among whites (APC, 3.06%; 95% CI, 0.50%–5.69%) but not blacks. Smoking prevalence decreased significantly among whites (APC, −1.99%; 95% CI, −3.86 to −0.09) but not blacks (Table 2).

## Discussion

Overall, we observed significant linear trends in APC from 2001 to 2010 in the prevalence of obesity, diabetes, and high cholesterol among Mississippi Delta adults; we also observed some racial differences. The increases in major CVD risks factors we found are similar to those reported in the 1991–1999 BRFSS (17). We also found that the prevalence of obesity and high cholesterol significantly increased among both blacks and whites, whereas the prevalence of diabetes significantly increased among blacks but not among whites. In addition, we observed a significant decrease in smoking prevalence among whites but not among blacks.

Mississippi had the highest prevalence of obesity in the nation in 2010 (18), and the Mississippi Delta had the highest rate in the state (7). The linear trends in obesity among blacks and whites found in our study are similar to those observed in the BRFSS and the National Health and Nutrition Examination Survey (NHANES) (17,19). Obesity is a risk factor for other risk factors and is linked to CVD (20); programs to prevent CVD and related chronic diseases, especially obesity, aimed primarily at children and adolescents may help to reverse the increases in obesity prevalence. Our findings also show that high cholesterol is rapidly increasing among blacks and whites in the Mississippi Delta. The trends in the prevalence of high cholesterol observed in our study are similar to trends shown by the BRFSS (17). Recent assessment of the prevalence of cholesterol screening and high cholesterol among all US adults in the BRFSS from 2005 to 2009 indicated that an increase in the prevalence of self-reported high cholesterol may be due to an increase in the awareness of the health risks posed by high cholesterol or an increase in the prevalence of high cholesterol among adults ever screened, or both (21). The increase in the prevalence of high cholesterol found in our study may be attributable to increasingly poor diets and increases in the consumption of high-calorie foods, coupled with rising obesity rates. Policies and interventions aimed at providing better access to affordable, healthful foods and low-fat diets may stem the rising trend (22,23).

High blood pressure is a major risk factor for heart disease and stroke (4); in 2012 it was the ninth leading cause of death in the Mississippi Delta (3). Despite high rates of high blood pressure in the Mississippi Delta (7,8), we did not observe significant increases in high blood pressure among the study population overall (blacks and whites combined). We may not have found a significant change in high blood pressure because we analyzed only 5 years of blood pressure data, compared with 10 years of data for all the other risk factors except high cholesterol; the longer the trend period, the more powerful the trend test.

In 2012, diabetes was the fourth leading cause of death in the Mississippi Delta (3). Our study found racial differences in the prevalence of diabetes: we found significant increases in the prevalence of diabetes among blacks but not among whites. Annual increases in diabetes prevalence among blacks were higher than increases in the prevalence of other risk factors. The trend among blacks observed in our study is similar to the trend found by using NHANES data on US adults 20 years or older (24). An increase in the prevalence of diabetes is likely related to heightened levels of disease detection (25) and increases in the prevalence of obesity (24). Diabetes can be a drain on the local economy as well: the indirect costs of diabetes include costs associated with absenteeism, reduced productivity among workers, increased disease-related disability, and lost work capacity due to early mortality (26).

Smoking prevalence significantly decreased among whites but not among blacks in the Mississippi Delta. Our results highlight that smoking prevention strategies and policies may not be reaching or are not successful in reducing smoking prevalence among black adults. There is a need for further investigation of the underling racial disparities in smoking prevalence in this region. Culturally targeted, sustained tobacco intervention policies and evidence-based cessation methods may help to reduce smoking rates among this population (27). The MDHC, in collaboration with mayors, established mayoral health councils to enact smoke-free polices in cities and towns throughout Mississippi Delta.

Our analyses showed a significant increase in physical inactivity among whites in the Mississippi Delta. In the 2010 BRFSS, 33.0% of Mississippi adults reported not participating in any physical activity in previous 30 days, with higher rates among blacks (38.7%) than among whites (30.0%) (7). Interventions aimed at increasing physical activity options for this population may help reverse this trend (22).

Our findings have several potential limitations. First, BRFSS consists of self-reported information on CVD risk factors and is prone to recall bias and the likelihood for socially desirable responses (eg, underreporting weight, indicating not currently smoking, overstating physical activity) (28). Second, the Mississippi Delta is a poor, underserved rural area, and many households may not have access or connectivity to a landline telephone; therefore, information collected during these survey periods may have underestimated the number of people with these risk factors (29). Third, we did not assess preventive measures, treatment, or control for these risk factors. Fourth, BRFSS survey methodology was not designed to provide an estimate of these risk factors at the regional level, although the distributions among respondents from 2001 to 2010 were similar and we believe that the 18-county region provided a sufficient sample size. Fifth, lack of access to health care in the Mississippi Delta might lead to underestimation of these risk factors.

The linear trends in CVD risk factors observed in this study have important public health implications because CVD is the leading cause of death in Mississippi Delta (2) and recent national data indicate that adjusted estimated population-attributable fractions for CVD mortality were 40.6% for high blood pressure, 13.7% for smoking, 13.2% for poor diet, 11.9% for insufficient physical activity, and 8.8% for abnormal blood glucose levels (4). Public health professionals, state and local policy makers, and stakeholders need to be aware of trends and annual increases in these factors to define achievable goals and monitor progress in reducing modifiable risk factors, including obesity.

The findings of this study provide useful information on linear trends and APCs for the population of the Mississippi Delta and could direct effective prevention strategies targeting modifiable risk factors for CVD. The Mississippi State Department of Health, in collaboration with CDC and other stakeholders, is currently implementing programs through the MDHC aimed at reducing heart disease, stroke, and associated risk factors in the Mississippi Delta (http://msdh.ms.gov/msdhsite/_static/44,0,372.html). Future studies should assess the effectiveness of the MDHC interventions in this region.
